# Supplementary material for: Characterization of Fructose-1,6-Bisphosphate Aldolase 1 of Echinococcus multilocularis
Source: Vet Sci. 2021 Dec 23;9(1):4. doi: 10.3390/vetsci9010004 (PMC8781991; doi:10.3390/vetsci9010004)
Supplement: Supplementary file 1 [file vetsci-09-00004-s001.zip › vetsci-1469986-supplementary.pdf]

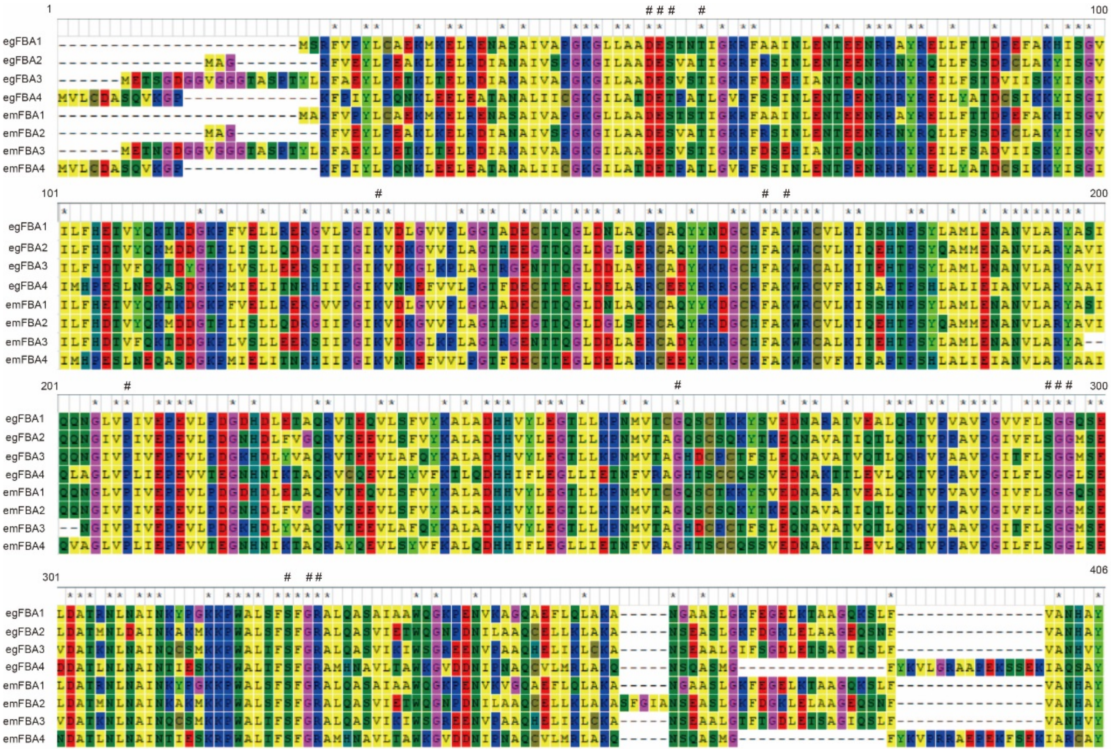

Figure S1. Alignment of the FBA amino acid sequences from *Echinococcus* species.
The numbers above the alignment represent the position of amino acids. The conserved amino acid residues were shown with '*', while the conserved active sites were shown with '#' above the alignment. The gaps in the alignment were filled with '-'. Amino acid sequences of FBA were retrieved from WormBase database: EmFBA1: Emu_J000905600, emFBA2: Emu_J000993700, emFBA3: Emu_J000993800, emFBA4: Emu_J000980100, egFBA1: Egr_G000905600, egFBA2: Egr_G000993700, egFBA3: Egr_G000993800, and egFBA4: Egr_G000980100.

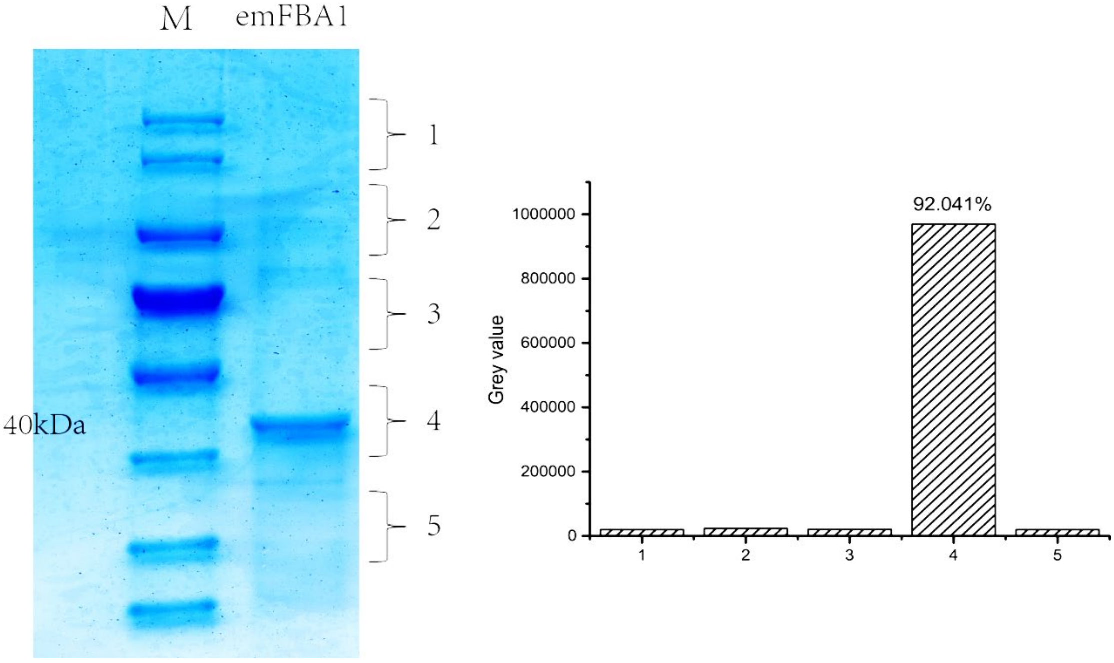

Figure S2. Purity analysis of EmFBA1. The greyscale intensity of 5 parts of the gel was analyzed by ImageJ. M: maker.

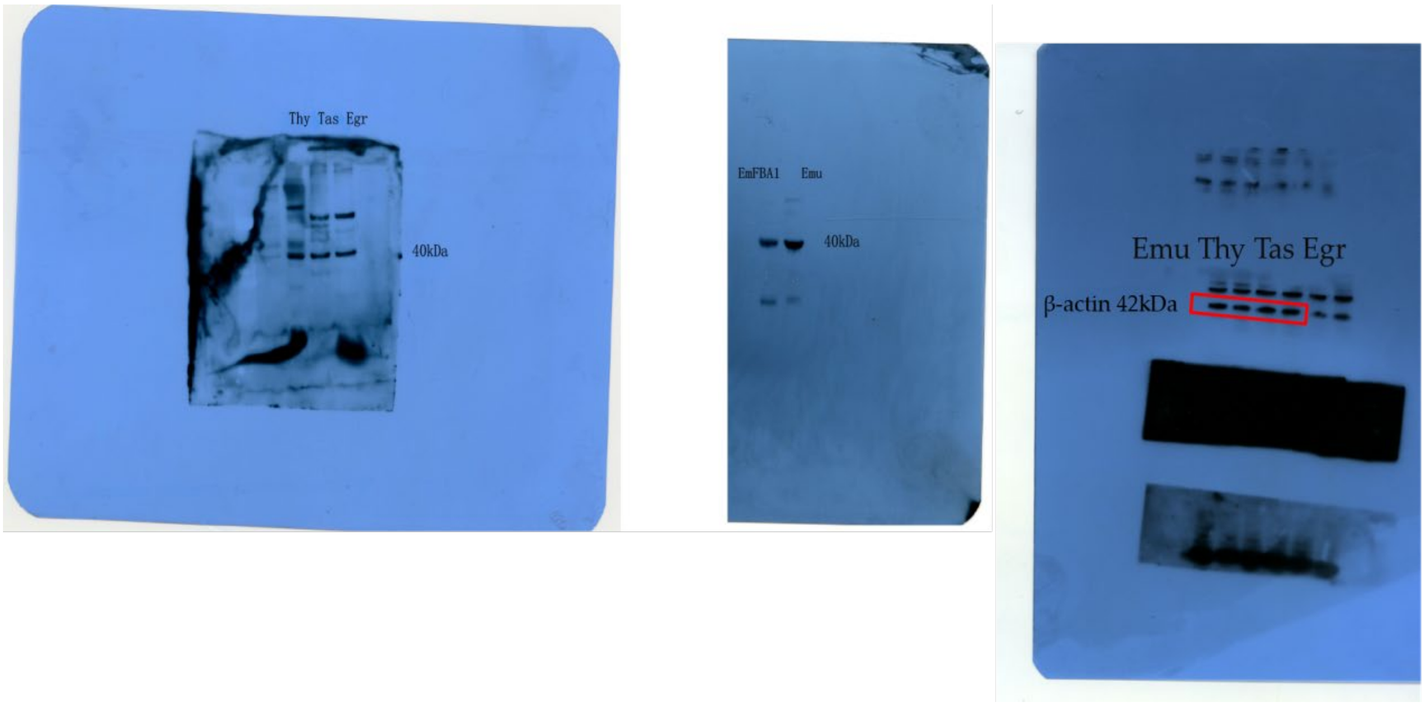

Figure S3. Western blotting analysis of natural FBA1 in *E. multilocularis* and other tapeworms. Emu, *E. multilocularis*; Egr, *E. granulosus*; Tas, *Taenia asiatica*; Thy, T*aenia hydatigena*.
